# Structure and function of a broad-specificity chitin deacetylase from *Aspergillus nidulans* FGSC A4

**DOI:** 10.1038/s41598-017-02043-1

**Published:** 2017-05-11

**Authors:** Zhanliang Liu, Laurie M. Gay, Tina R. Tuveng, Jane W. Agger, Bjørge Westereng, Geir Mathiesen, Svein J. Horn, Gustav Vaaje-Kolstad, Daan M. F. van Aalten, Vincent G. H. Eijsink

**Affiliations:** 1 Faculty of Chemistry, Biotechnology, and Food Science, The Norwegian University of Life Sciences, Ås, 1432 Norway; 2 College of Life Sciences University of Dundee, Dow Street, Dundee, DD1 5EH Scotland United Kingdom

## Abstract

Enzymatic conversion of chitin, a β-1,4 linked polymer of *N*-acetylglucosamine, is of major interest in areas varying from the biorefining of chitin-rich waste streams to understanding how medically relevant fungi remodel their chitin-containing cell walls. Although numerous chitinolytic enzymes have been studied in detail, relatively little is known about enzymes capable of deacetylating chitin. We describe the structural and functional characterization of a 237 residue deacetylase (*An*CDA) from *Aspergillus nidulans* FGSC A4. *An*CDA acts on chito-oligomers, crystalline chitin, chitosan, and acetylxylan, but not on peptidoglycan. The *K*_m_ and *k*_cat_ of *An*CDA for the first deacetylation of penta-*N*-acetyl-chitopentaose are 72 µM and 1.4 s^−1^, respectively. Combining mass spectrometry and analyses of acetate release, it was shown that *An*CDA catalyses mono-deacetylation of (GlcNAc)_2_ and full deacetylation of (GlcNAc)_3–6_ in a non-processive manner. Deacetylation of the reducing end sugar was much slower than deacetylation of the other sugars in chito-oligomers. These enzymatic characteristics are discussed in the light of the crystal structure of *An*CDA, providing insight into how the chitin deacetylase may interact with its substrates. Interestingly, *An*CDA activity on crystalline chitin was enhanced by a lytic polysaccharide monooxygenase that increases substrate accessibility by oxidative cleavage of the chitin chains.

## Introduction

Chitin is an abundant insoluble natural polysaccharide comprised of β-1,4-linked *N*-acetyl-D-glucosamine residues, that is found in the cell walls of fungi and some algae, and in the exoskeletons or cuticles of many invertebrates. In Nature, a plethora of enzymes act on chitin, in particular a wide variety of depolymerizing enzymes (e.g. chitinases^1, 2^) as well as chitin-deacetylases, which release acetate from the acetamido group at C2, generating D-glucosamine^3, 4^.

Deacetylation of chitin is an interesting process because it affects the crystallinity and solubility of the polymer, which may be of importance during growth and morphogenesis of chitin-containing organisms. Several studies have shown that chitin deacetylases (CDAs) modulate chitin-rich fungal cell walls during growth and cell division^5–7^. Deacetylation of chitin in the cell walls of plant pathogenic fungi may affect virulence^5, 8, 9^ because deacetylation reduces the susceptibility of the fungal chitin to degradation by chitinases that are secreted as part of the plant’s defence response. Furthermore, deacetylation of chitin fragments released from the fungal cell wall may reduce the potential of these fragments to elicit plant defence responses^10^. On the applied side, products of chitin deacetylation and depolymerisation, i.e. chitosans with different degrees of polymerization and acetylation, have several interesting functionalities^11, 12^.

Chitin deacetylases belong to the family 4 of carbohydrate esterases (CE4), according to the CAZy classification system (www.cazy.org ^13^). This esterase family comprises enzymes that de-*N*- or de-*O*-acetylate chitin, acetyl xylan and peptidoglycan. Deacetylases in this family share a universal conserved region, namely the NodB homology domain^14^, which, for active deacetylases includes five conserved sequence motifs with conserved aspartic acid and histidine residues and a conserved binding site for a catalytically important metal ion^9, 15–18^. Putative members of the CE4 family are abundant in the genomes of chitin-containing fungi (www.cazy.org), indicating that these enzymes have important biological roles. Notably, both chitin deacetylases and peptidoglycan deacetylases are interesting targets for the development of antimicrobial agents^19^, underpinning the importance of obtaining a better understanding of the enzymatic mechanism and structure of these enzymes.

Although several putative CDAs have been described in the literature, only few have been studied in detail, including CDAs from *Mucor rouxii* ^20, 21^, *Colletotrichum lindemuthianum* ^9, 22^, *Puccinia graminis* ^23^, *Vibrio parahaemolyticus* ^24^ and *Vibrio cholerae* ^18^. The genome of *Aspergillus nidulans* FGSC A4^25^ contains six genes putatively encoding polysaccharide deacetylases in the CE4 family. Previous work^26^ has shown that *Aspergillus nidulans* secretes chitin deacetylases. More recently, Wang *et al*.^27^ described the cloning and preliminary characterization of a putative CDA from *A. nidulans* AF93062. We have cloned a similar chitin deacetylase (EAA66447) from *Aspergillus nidulans* FGSC A4 and carried out an extensive characterization of the recombinant enzyme, with an appended short N-terminal His-tag, produced in *E. coli*. We have determined the crystal structure of *An*CDA and analysed enzyme activity on a variety of substrates. Insight into preferred substrate-binding modes of the enzyme was obtained by mass spectrometry-based sequence analysis^28^ of partially deacetylated oligosaccharide products. The activity data were interpreted using the novel crystal structure with and without a docked substrate and by comparing both the activity and structural data with available data for other chitin deacetylases. Thus, novel insights into potential structure-function relationships in family CE4 CDAs were obtained. We also show that the activity of *An*CDA on insoluble chitin is boosted by co-administration of CBP21 a chitin-active lytic polysaccharide monooxygenase (LPMO^29^) from *Serratia marcescens*.

## Results and Discussion

### A. nidulans possesses three canonical chitin deacetylases

**Figure 1:**
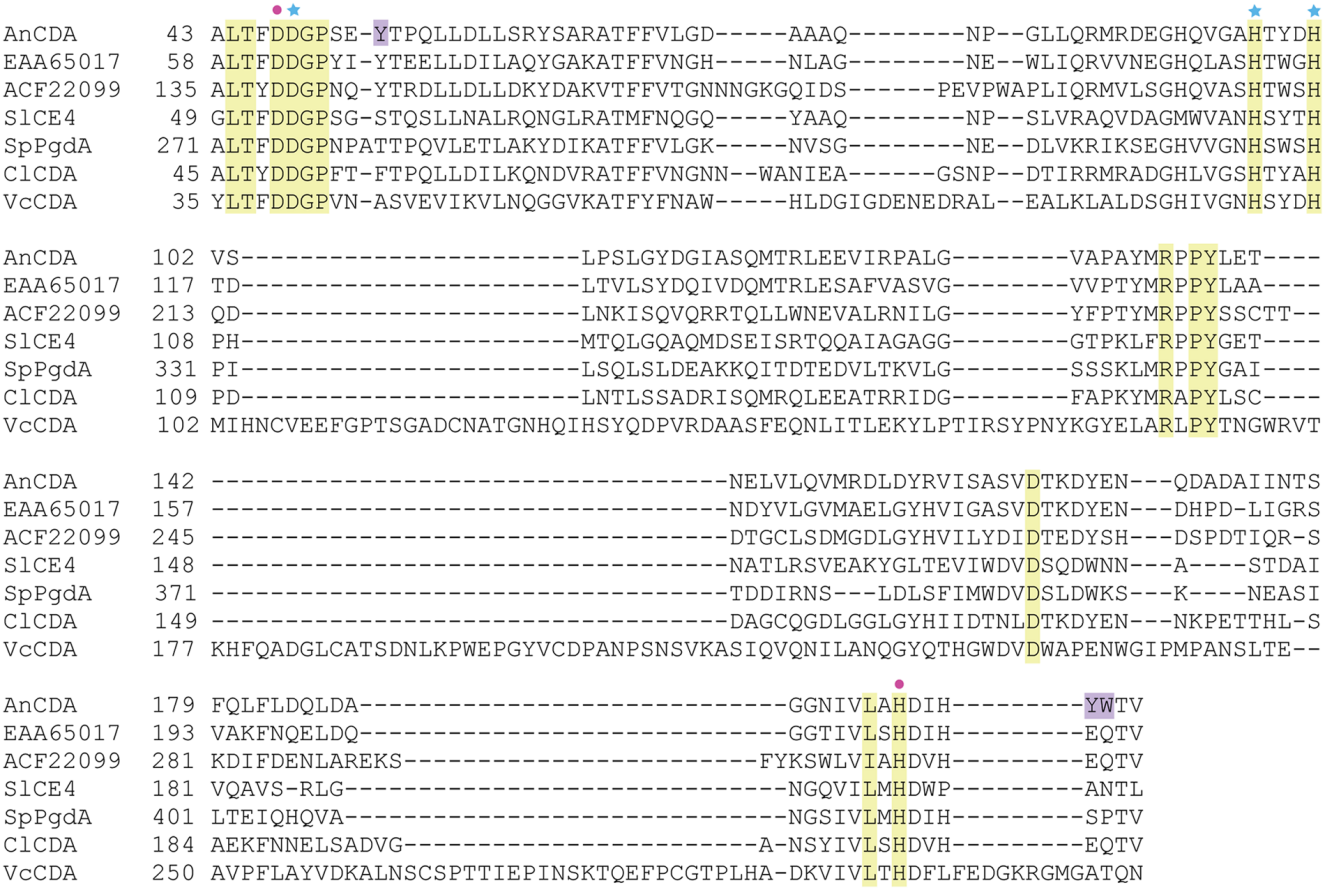
Structure-based multiple sequence alignment of the catalytic (NodB-like) domains of CE4 family enzymes. The alignment comprises four enzymes with known deacetylase activity and three putative chitin deacetylases from *Aspergillus nidulans* FGSC A4, *An*CDA (EAA66447, this study), EAA65017 and ACF22099. The other enzymes are: *Sl*CE4, acetyl xylan deacetylase from *Streptomyces lividans*, AAC06115.2^17^; *Sp*PgdA, peptidoglycan deacetylase from *Streptococcus* *pneumoniae*, NP_358926^15^; *Cl*CDA, CDA from *Colletotrichum lindemuthianum*, AAT68493^9^; *Vc*CDA, CDA from *Vibrio cholerae*, AAF94439.1^18^. Fully conserved residues in the five sequence motifs that are important for activity (see text)^15^ are shown with yellow background. The purple background indicates aromatic surface residues in *An*CDA that are discussed in the text. Blue asterisks indicate residues in the metal binding triad, while pink dots indicate the catalytic acid and base^15, 17^. The alignment was prepared with PyMod 2.0 (plugin in PyMol)^51^ by doing a structure based sequence alignment of *An*CDA, *Sl*CE4, *Sp*PgdA, *Cl*CDA, and *Vc*CDA, before adding EAA65017 and ACF22099 to the alignment.

The genome of *Aspergillus nidulans* FGSC A4 encodes six putative polysaccharide deacetylases belonging to the CE4 family (www.cazy.org/fam/CE4.html). Sequence alignments revealed that three of these (EAA66447, ACF22099, EAA65017) contain the five distinct sequence motifs (TfDDGP, Hs/txxHp, Ra/pPY, DxxDw/y, and ivlxHd/e) that are conserved in CE-4 family members with documented deacetylase activity (Fig. 1)^15^. One of these enzymes has two additional chitin-binding domains (ACF22099), whereas the remaining two are single domain proteins comprising only a CE4 catalytic domain (domain annotation according to www.pfam.org and www.uniprot.org). In this study, we characterized the single domain protein, EAA66447, designated *An*CDA.

The *An*CDA gene contains two introns and encodes a primary gene product of 237 amino acid residues. The primary gene product contains an N-terminal signal sequence which is predicted to be removed by cleavage between Thr18 and Thr19 (http://www.cbs.dtu.dk/services/SignalP)^30^. The coding regions, excluding the signal sequence, were cloned by overlap extension polymerase chain reactions and inserted into the pBAD/HisB(s) vector, after which the protein was expressed in *E. coli* TOP10 (for all enzymology work) or *E. coli* BL21 (for crystallography). The protein, containing a short N-terminal His-tag, was purified to homogeneity by Ni^2+^ affinity chromatography and used for further studies. Purity was confirmed by SDS-PAGE analysis.

### AnCDA is a deacetylase with broad substrate specificity

Standard assays based on the detection of released acetate by ion chromatography (IC) showed that purified *An*CDA was active towards (GlcNAc)_6_. The activity of CE4 deacetylases depends on bivalent metals^15, 17^, and previous experiments as well as our own initial characterizations showed that of several bivalent metal ions tested, Co^2+^ was the most beneficial for activity. For this reason, all enzyme reactions contained Co^2+^. Studies of the effect of temperature and pH on activity towards (GlcNAc)_6_ showed optima of approximately 50 °C (broad optimum) and pH 8.0 (narrow optimum), respectively. Pre-incubation studies showed that *An*CDA is stable for one hour at temperatures between 30 and 60 °C (pH = 8.0) and pH 6.0–10.0 (T = 37 °C). Based on these results, all further reactions were carried out at 37 °C and pH 8.0, unless noted otherwise.

Additional assays showed that *An*CDA is active towards a variety of substrates including (GlcNAc)_2–6_ (Table 1). Long incubations with high enzyme concentrations showed that *An*CDA is inactive towards the GlcNAc monomer and catalyses mono-deacetylation of (GlcNAc)_2_. For longer oligomeric substrates, fully deacetylated products were produced at a very low rate (see below for further details).

**Table 1:** Activity of *An*CDA.

Substrate	Concen-tration	ASAR (mM)^a^	Average acetic acid release (µM)	CV%^b^	Acetic acid released (nmol/min)	Apparent rate constant (s^−1^)	Deacetylation degree after 15 min (%)
GlcNAc	2 mM	2	N.D.				
(GlcNAc)_2_	2 mM	4	13.2	18	0.09	0.2	0.3
(GlcNAc)_3_	2 mM	6	43.0	11	0.29	1.2	0.7
(GlcNAc)_4_	2 mM	8	16.1	13	0.11	0.5	0.2
(GlcNAc)_5_	2 mM	10	56.0	3	0.37	1.6	0.6
(GlcNAc)_6_	2 mM	12	43.5	8	0.29	1.2	0.4
Chitosan^c^	5 mg/mL	16	87.8	9	0.59	2.4	0.6
Acetyl xylan	5 mg/mL	9^d^	308	12	2.1	8.6	3.4
α-chitin	5 mg/mL	24.6^e^	7.9	17	0.05	0.2	0.03
β-chitin	5 mg/mL	24.6^e^	21.5	9	0.14	0.6	0.09

All of the substrates were incubated with 40 nM *An*CDA in a 100 μL reaction volume for 15 minutes at standard reaction conditions. Note that under these conditions, the degree of deacetylation is very low, which implies that multiple deacetylations of oligomeric substrates are highly unlikely (see also below). ^a^ASAR, substrate concentration expressed as the concentration of acetyl groups. ^b^CV%, coefficient of variance. ^c^Solubilized chitosan with degree of N-acetylation of 64% (F_A_ = 0.64), with a random distribution of *N*-acetylated and de-*N*-acetylated units. ^d^The acetate concentration was estimated after complete deacetylation with 50 mM aqueous NH_3_ over night at 4 °C. ^e^Assuming one acetylation per sugar unit.

**Figure 2:**
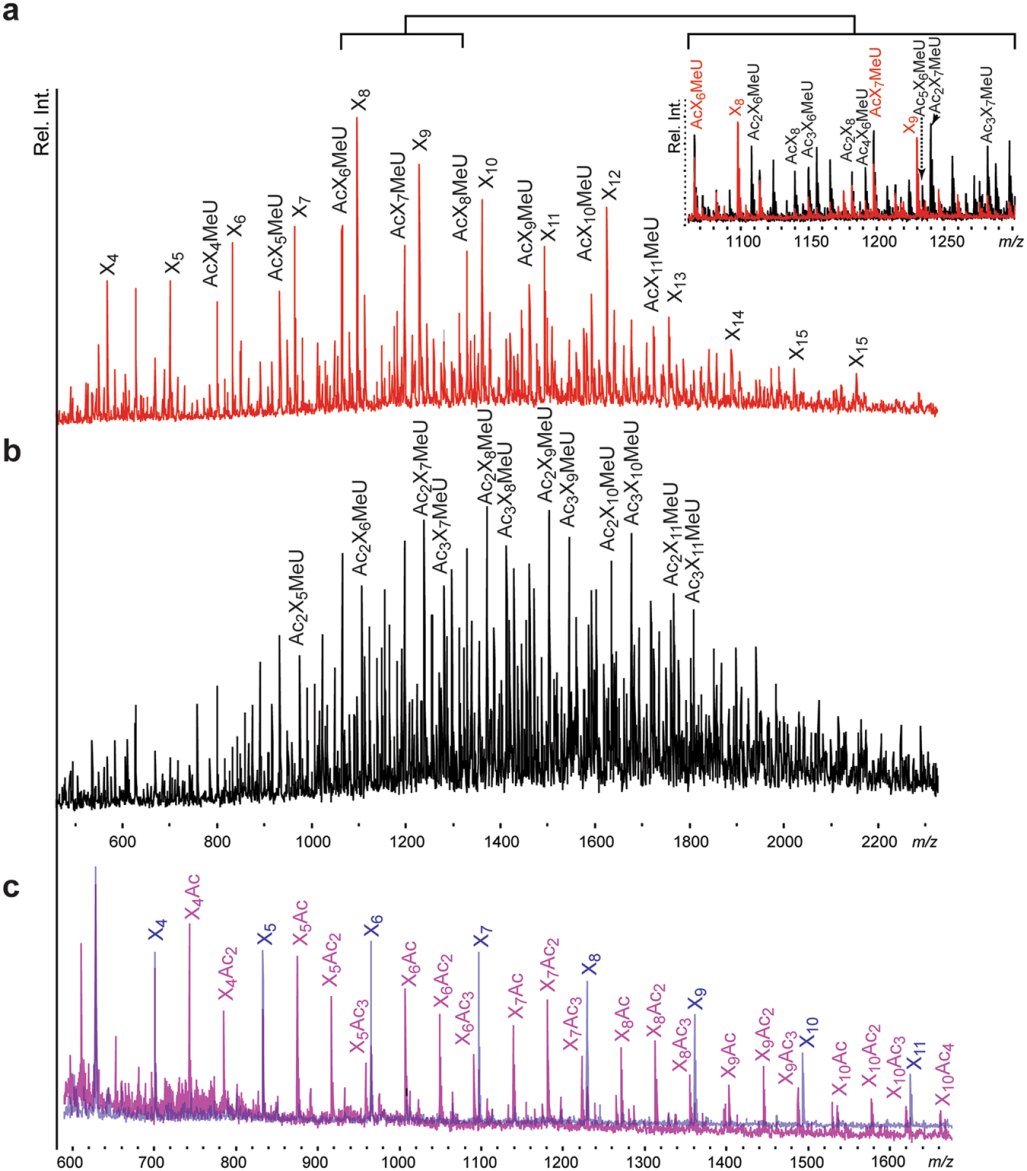
Activity of *An*CDA on acetylated glucuronoxylan (**a**,**b**) and acetylated xylan (**c**). The pictures show MALDI-ToF spectra of oligosaccharides. Peaks are labelled by sugar composition; X, xylose; Ac, acetyl group; MeU, 4-O-methylglucuronic acid. Panel a, acetylated glucuronoxylan after treatment with *An*CDA; panel b, untreated acetylated glucuronoxylan; insert in panel a, comparison of panels a and b; panel c, acetylated xylan before (pink) and after (blue) treatment with *An*CDA.

*An*CDA showed no activity on peptidoglycan, but the enzyme was active on oligomeric acetylxylan and acetylated glucuronoxylan, as well as on chitosan with an initial fraction of acetylated residues (F_A_) of 0.64 (Table 1). MALDI-ToF MS analysis of the acetylated xylans before and after extensive enzyme treatment showed that the enzyme reduced the complexity of the oligosaccharide mixtures. In the case of acetylated glucuronoxylan the dominating products were fully deacetylated xylooligosaccharides and single acetylated xylooligosaccharides containing one 4-O-methylglucuronic acid (Fig. 2a,b). In the case of acetylated xylan, the dominating products were fully deacetylated xylooligosaccharides (Fig. 2c). A six-day incubation of *An*CDA with chitosan (F_A_ = 0.61), using 4 µg enzyme per gram of substrate, at 50 °C in 100 mM Tris-HCl, pH 8.0 and 2.5 µM CoCl_2_ changed the fraction of de-*N*-acetylated sugars in the chitosan (F_A_) from 0.61 to 0.09 (as determined by NMR^31^).

It is has been shown previously that almost all CE4 enzymes, including the peptidoglycan deacetylases, can deacetylate chito-oligomers^14–16, 32, 33^. Furthermore, it has been shown that CE4 members classified as acetylxylan esterases can deacetylate chitosan and chito-oligomers^14, 16, 17, 34^. However, comparative information on rates is scarce. Puchart *et al*. (2006) used initial-rate conditions to characterize an acetylxylan esterase from *Streptomyces lividans* and found that this enzyme is several orders of magnitude more active towards acetylated xylan than towards chito-oligomers^16^. Similar observations were made for an acetylxylan esterase from *Clostridium thermocellum* ^17^. Thus, there is a clear difference between the deacetylase described here, acting almost equally well on chito-oligomers, chitosan and acetylxylan (Table 1), and these previously studied acetylxylan esterases.

An interesting feature of the data in Table 1 is the somewhat counter-intuitive relationship between the deacetylation rate and the length of chito-oligomeric substrates, which is most visible in the fact that (GlcNAc)_4_ is deacetylated at a clearly lower rate than both (GlcNAc)_3_ and (GlcNAc)_5_. This is discussed below.

### AnCDA is active on insoluble chitin

Published studies on chitin deacetylases report only low activities towards crystalline chitin^14, 32, 35^, with typical maximum degrees of deacetylation in the order of 0.5%. Similar observations were made for *An*CDA. In the initial rate type of assay depicted in Table 1, 40 nM *An*CDA released only 0.03% and 0.09% of the acetyl groups from 5 mg/ml α-chitin and β-chitin in 15 min, respectively. Incubation with 1 μM *An*CDA for 24 h released approximately 0.5% of the acetyl groups from 1 mg/ml of α-chitin or β-chitin.

**Figure 3:**
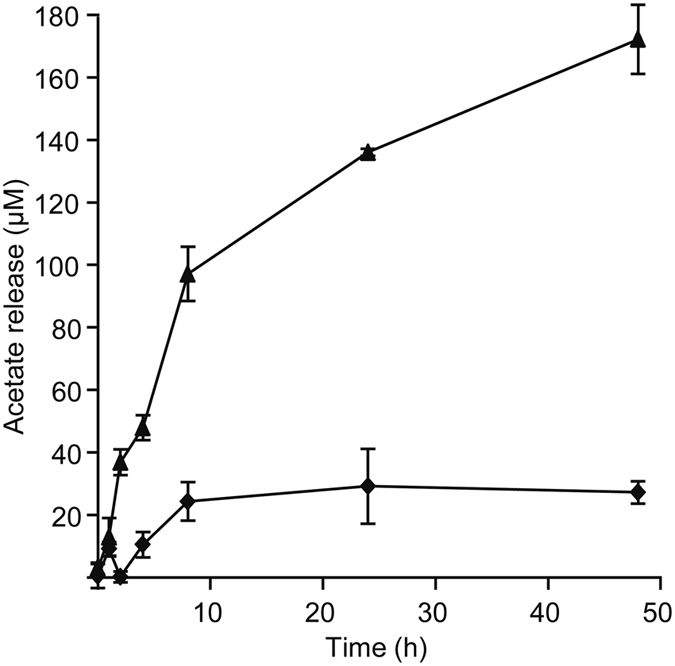
Effect of CBP21 on deacetylation of β-chitin by *An*CDA. 1 mg/mL β-chitin was incubated with 1 μM *An*CDA (diamonds) or with 1 μM *An*CDA and 1 μM CBP21 (triangles) in 50 mM Tris-HCl, pH 8.0 containing 250 µM CoCl_2_ and 1 mM ascorbic acid at 37 °C. In a control reaction with only 1 μM CBP21 no acetate release was observed. Note that acetate release after 24 hours in the absence of CBP21 corresponds to approximately 0.5% deacetylation.

The low activity of CDAs on insoluble chitin is likely due to poor accessibility of the substrate. Indeed, more accessible substrates, such as chitosan and amorphous chitin, are normally deacetylated more efficiently, as demonstrated above and in previous studies^14, 32, 34, 35^ (note that partially de-acetylated soluble chitin-forms are collectively referred to as chitosan in the present paper, but appear in older literature under varying names). Recently, Vaaje-Kolstad *et al*.^29^ have discovered that proteins previously classified as CBM33 in the CAZy database^13^ are enzymes that cleave chitin chains in their crystalline context, using an oxidative mechanism^29^. These lytic polysaccharide monooxygenases (LPMOs) boost the apparent activity of chitinases by improving access to the substrate. Figure 3 shows that CBP21, a chitin-active LPMO from *S. marcescens*, boosted the activity of *An*CDA. *A. nidulans* encodes 13 LPMOs and the observed synergistic effect seen between *An*CDA and *Serratia* LPMO could thus be biologically relevant.

### In-depth studies of the deacetylation of (GlcNAc)_5_ and (GlcNAc)_6_

**Figure 4:**
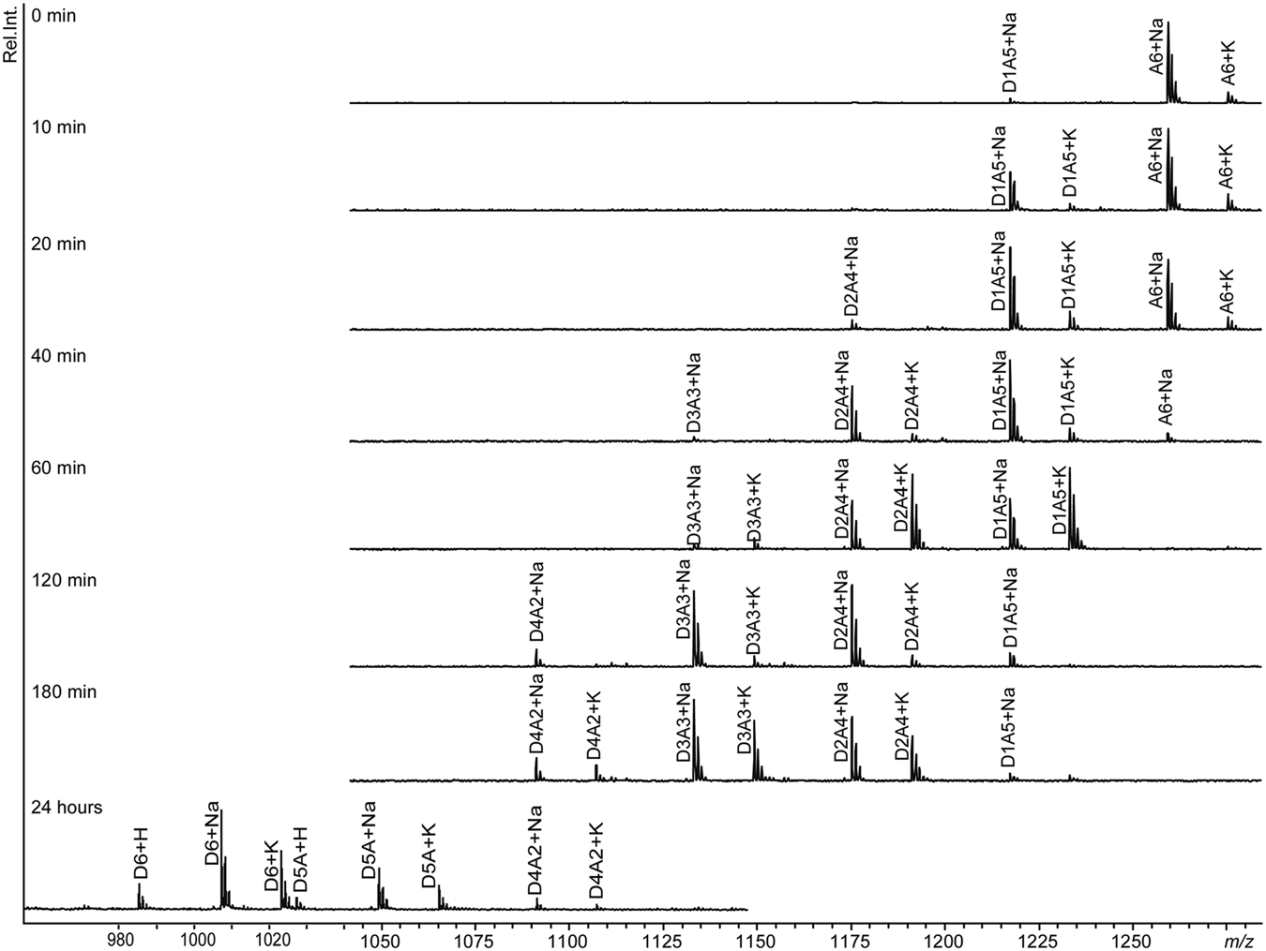
Product profiles during deacetylation of (GlcNAc)_6_ by *An*CDA. The reaction mixture contained 2 mM (GlcNAc)_6_, 400 nM *An*CDA, and 10 μM CoCl_2_ in 50 mM Tris pH 8.0 and was incubated at 37 °C. The Figure shows annotated MALDI-ToF spectra of the generated produced mixtures at various time points during the reaction. The 24 hours sample is from a similar reaction, with higher (1 µM) enzyme concentration.

To analyse the progress of the enzymatic deacetylation reaction over a longer time period MALDI-ToF MS was used to trace intermediate products. Reaction mixtures contained 2 mM (GlcNAc)_6_, 10 μM CoCl_2_, 50 mM Tris (pH 8.0), and 400 nM *An*CDA. Product profiles (Fig. 4) showed that, initially, the A_6_ substrate is almost exclusively converted to A_5_D_1_. Only after the A_6_ signal has become almost undetectable do products with higher degrees of deacetylation become clearly visible. This indicates that *An*CDA prefers fully acetylated chito-oligosaccharides, rather than partially deacetylated species. On the other hand, Fig. 4 shows that prolonged incubation with high enzyme doses yielded the fully deacetylated product, (GlcN)_6_ (D_6_).

**Figure 5:**
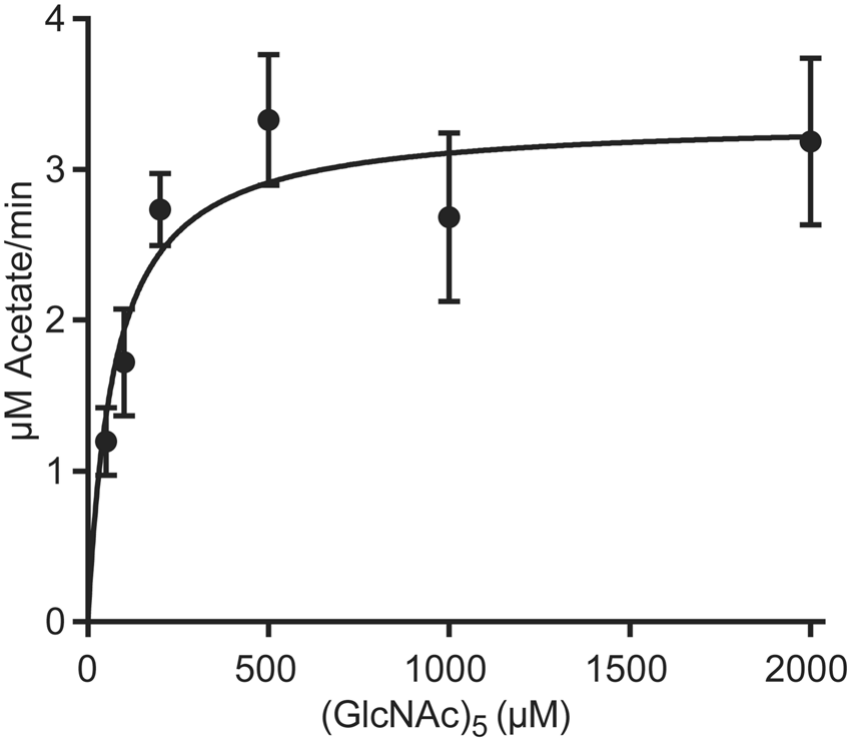
Steady-state kinetics of *An*CDA with (GlcNAc)_5_. The Figure shows experimental data points with standard deviations and the theoretical fit to these points determined by hyperbolic regression.

Michaelis-Menten analysis was carried out using the best substrate, (GlcNAc)_5_, under similar conditions, and with only 15 minutes incubation time (Fig. 5). This analysis yielded *K*_m_ and *k*_cat_ values of 0.072 +/− 0.022 mM (i.e. 0.36 mM acetyl groups) and 1.4 +/− 0.1 s^−1^, respectively. These kinetic parameters are similar to those found for *Cl*CDA acting on (GlcNAc)_5_ (*K*_m_ = 0.08 mM, *k*_cat_ = 7 s^−1^)^22^. Kinetic information for other CDAs is limited, but several *K*_m_ values have been reported that are generally one order of magnitude higher than the values found for *An*CDA and *Cl*CDA^35^.

**Figure 6:**
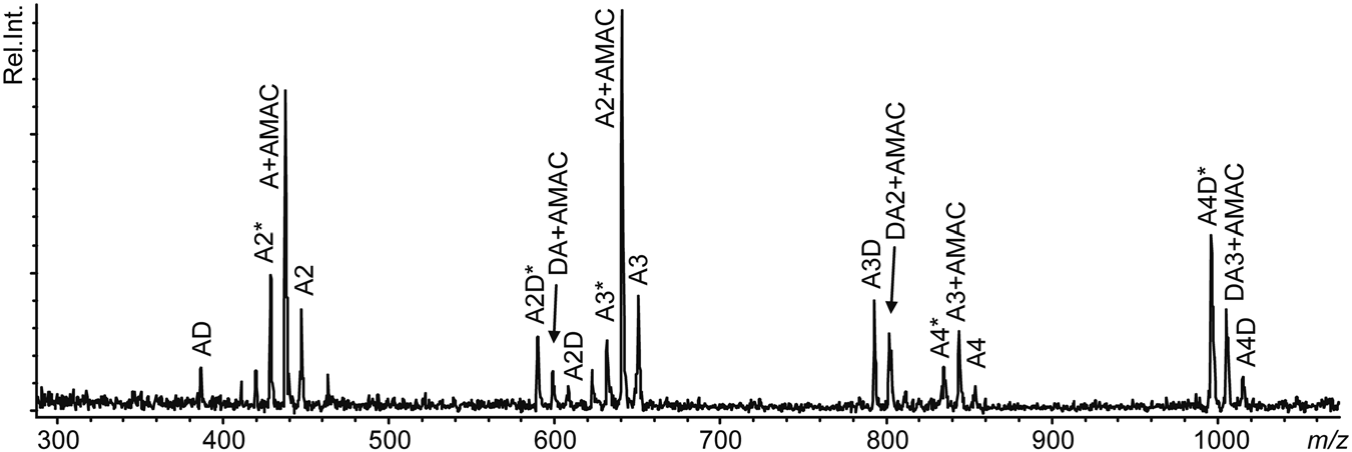
MALDI-ToF MS/MS analysis of AMAC-labelled D_1_A_5_. Several compounds occur in several forms (i.e. multiple masses), due to the loss of water (labelled *) during fragment formation. Abbreviations: A, *N*-acetylglucosamine; D, glucosamine; AMAC, 2-aminoacridone.

The product patterns shown in Fig. 4 imply that *An*CDA hydrolyses only one acetyl-group per substrate-binding event and thus is non-processive. To gain further insight into the preferred productive binding modes of *An*CDA, deacetylation products generated from (GlcNAc)_6_ were labelled with 2-aminoacridone (AMAC), fragmented and analysed by MALDI-ToF-MS with post-source decay. MS/MS data for the AMAC-labelled D_1_A_5_ product are shown in Fig. 6. The spectrum shows a variety of molecules including A-AMAC, A_2_-AMAC, A_3_-AMAC and DA-AMAC, but no compounds with -D-AMAC. These results show that the first deacetylation takes place at varying positions, but not at the reducing end. Similar analysis of other partially deacetylated hexamers confirmed that deacetylation occurs at random positions, except for the reducing end. The fact that the acetamido group of the reducing end sugar is hydrolysed much slower than the others was clearly confirmed by these analyses: A-AMAC was detected after fragmentation of all of the AMAC-labelled partially deacetylated oligomeric products (i.e. D_1_A_5_, D_2_A_4_, D_3_A_3_, D_4_A_2_, and D_5_A_1_), whereas D-AMAC was not observed. The MS data were not conclusive as to whether the first deacetylation could occur at the non-reducing end.

### *An*CDA possesses a conserved substrate binding groove and a surface that seems adapted to binding longer substrates

**Figure 7:**
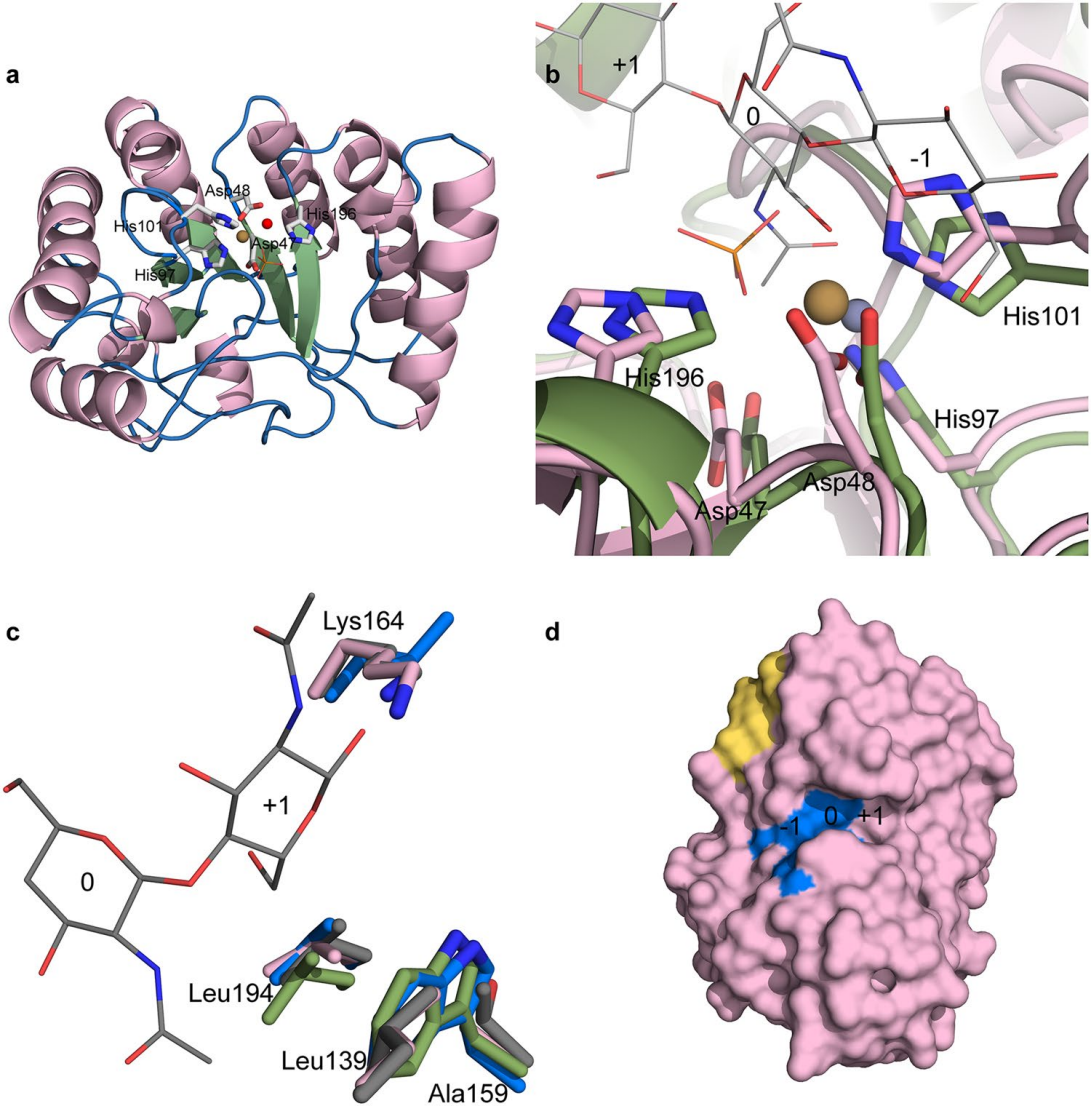
Crystal structure of *An*CDA. (**a**) Structure of *An*CDA coloured by secondary structure; green = strand, pink = helix and blue = loop/coil. The side chains of the metal binding residues, the catalytic acid and the catalytic base are labelled and shown as sticks, the phosphate ion is shown as orange lines, and the metal ion is shown as a brown sphere. The water ion participating in the octahedral arrangement of the metal is shown as a red sphere. (**b)** Structural superposition of *A*nCDA (pink) and *Vc*CDA (green; PDB id: 4OUI)^18^ with a (GlnNAc)_3_ (grey lines) in the active site. The side chains of the metal binding His-His-Asp triad and the catalytic acid and base are shown as sticks, with numbering according to *An*CDA. The phosphate ion in the *An*CDA structure is shown as orange sticks. Subsites are labelled −1, 0, and +1. The brown sphere is the Co^2+^ ion bound in *An*CDA, while the dark grey sphere is the Zn^2+^ ion bound in *Vc*CDA. The structural superposition was generated using the PyMod 2.0^51^ plugin in PyMol. (**c**) Structural differences around subsite +1 in *An*CDA (pink), *Vc*CDA (green), *Sp*PgdA (blue; PDB id: 2C1G)^15^, and *Cl*CDA (grey; PDB id: 2IW0)^9^, showing the sidechains (in sticks) of the residues forming the hydrophobic pocket and the lysine special for *An*CDA and *Cl*CDA (see text). The ligand is shown as thin lines with grey carbons. At the position of Leu139 in *An*CDA, *Cl*CDA also has a leucine (shown), while *Sp*PgdA has a glycine and *Vc*CDA has a threonine (both not shown). At the position indicated by Ala159 in *An*CDA, *Cl*CDA has a threonine whereas the other two CDAs have a tryptophan (all shown). At the position indicated by Leu194 in *An*CDA, the other three CDAs also have leucine (all shown). At the position indicated by Lys164 in *An*CDA, *Cl*CDA also has a lysine, whereas *Sp*PgdA and *Vc*CDA have leucine (shown) and alanine (not shown), respectively. (**d)** Surface representation of *An*CDA, showing the active site residues (H97, H101, D48, D47, H196) in blue with subsite numbering. The yellow area corresponds to the hydrophobic patch (Y53, Y200, W201) discussed in the text.

To characterize the nature of the substrate-binding site in *An*CDA, we determined the crystal structure of the enzyme to 2.0 Å resolution (Table 2). The structure shows the expected (β/α)_8_ barrel topology as well as a CE4 active site architecture, including the His-His-Asp metal-binding triad (H97, H101, D48), a catalytic acid (His196, aiding sugar departure) and a catalytic base (Asp47, activating the nucleophilic water), similar to the active site of *Cl*CDA^9^ (Fig. 7a). Despite the fact that no metals were added in the crystallization solution, there is clear evidence for a metal ion bound to the His-His-Asp triad, with a peak >14 σ in the 2F_o_-F_c_ map. The ion, refined as cobalt, adopts an octahedral coordination in which the other three ligands are a bidentate phosphate molecule from the crystallization solvent and a water (Fig. 7a).

**Table 2:** Crystallographic data. Values in parentheses are for the highest resolution shell.

	*An*CDA (PDB ID: 2Y8U)
Space Group	P 2_1_
Wavelength (Å)	0.9763
Resolution (Å)	50–2.0 (2.1–2.0)
Cell dimensions (Å)	
*a*	35.64
*b*	64.52
*c*	86.54
Beta	101.16°
Total Reflections	94878 (13881)
Unique reflections	26431 (3855)
Completeness (%)	99.7 (99.8)
R_sym_	0.082 (0.316)
I/σI	10.3 (3.9)
Redundancy	3.6 (3.6)
R_cryst_	0.166
R_free_	0.212
RMSD from ideal bonds	0.023
RMSD from ideal angles	1.944
B-factor average	15.15
Ramachandran (coot)	
Favored	95.23%
Allowed	3.58%
Not so allowed	1.19%

**Figure 8:**
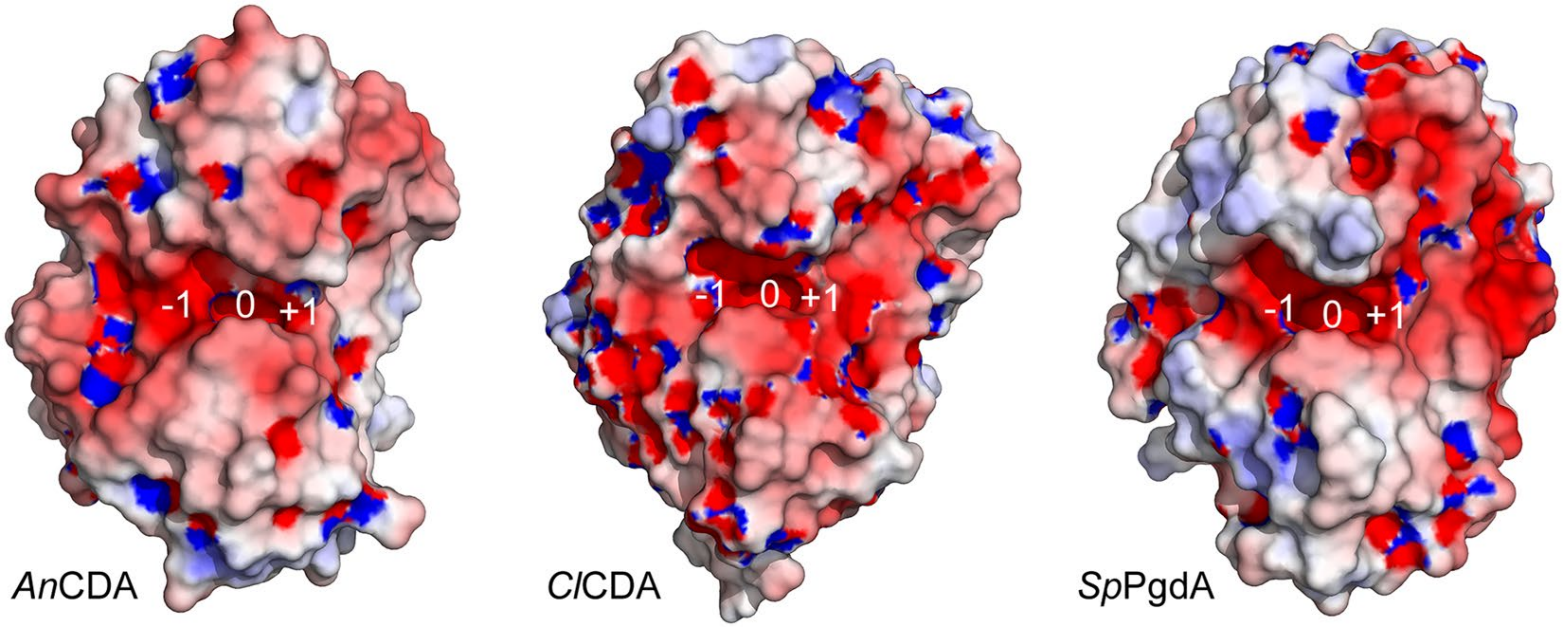
Comparison of charge distributions in deacetylases. The pictures show the surfaces of *An*CDA, *Cl*CDA (PDB id: 2IW0^9^), and *Sp*PgdA (PDB id: 2C1G^15^) coloured by charge calculated using the ABPS^52^ plugin in PyMol. Red represents negative charge and blue represents positive charge; solvent accessible surface potentials were set to −5 and 5 kT/e. The approximate locations of subsites −1, 0, and +1 are indicated.

Structural superposition of *An*CDA with the structure of a *Vc*CDA in complex with chitotriose provided insight in possible substrate binding (Fig. 7b). The phosphate ion in *An*CDA occupies the position where the acetyl-group of the to-be-deacetylated sugar, i.e. the sugar bound in subsite 0, is located. The organization of subsite 0 is very similar in the two enzymes. The −1 sugar can make several interactions with fully conserved elements of the catalytic machinery (Asp47, His101). The sugar bound in subsite +1 (Fig. 7c) interacts with Leu139 and Leu194 that form a hydrophobic pocket, similar as for *Cl*CDA^9^. Interestingly the corresponding pocket in bacterial CDAs contains an aromatic amino acid at a position corresponding to Ala159 in *An*CDA (Fig. 7c), whereas in these bacterial enzymes the leucine at position 139 in *An*CDA is replaced by glycine. Apparently, hydrophobic surface is important in subsite +1, and Nature has found several solutions to this. Interestingly, *An*CDA (and *Cl*CDA) have a lysine (Lys164 in *An*CDA) within hydrogen-bonding distance to the sugar bound in subsite +1, creating a positively charged environment (Fig. 8). This could reduce the affinity for deacetylated sugars in this subsite as the amino group of such a sugar carries positive charge. The AMAC-labelling results show that the reducing end is deacetylated at very low rate, indicating that binding of a sugar in subsite +1 is important for effective catalysis. It is possible that this sugar preferably should be acetylated. It should be noted, however, that the pH used in the present studies (8.0) was higher than the p*K*_a_ of the amino group in glucosamine (approximately 6.5^36^), reducing the potential charge effects discussed above.

The counter intuitive apparent rate constants presented in Table 1, showing higher rates for short odd-numbered chito-oligomers compared to the even-numbered chito-oligomers imply that there could be more subsites in *An*CDA that are not visible in or can be predicted from currently available structural information for members of the CE4 family. We have no straightforward explanation for the kinetic observations with chito-oligomers. If there are more than three subsites, it is possible that there are non-productive binding modes that may vary depending on chito-oligomer length in a counter-intuitive manner. For example, one could imagine that a trimer always binds productively, whereas a tetramer could interact with a more remote part of the enzyme surface, which could lead to a non-productive orientation of the substrate. Adding yet another sugar could lead to additional interactions favouring productive orientations.

Another interesting feature of the *An*CDA structure is the presence of a cluster of large aromatic residues on the surface of the protein near the catalytic centre and the −1 subsite (Fig. 7d). This cluster is composed of Tyr53, which is a phenylalanine in *Cl*CDA and tyrosine in all three putative *A. nidulans* deacetylases, and Tyr200 and Trp201, which are unique to *An*CDA (Fig. 1). Exposed aromatic residues are known to play roles in substrate binding by carbohydrate-processing enzymes, and may aid *An*CDA in binding longer chito-oligomers. Another possible role of these aromatic residues could be that they contribute in binding to the surface of an insoluble, and perhaps even crystalline, polysaccharide substrate, thus providing a similar functionality as the carbohydrate-binding modules that occur in many carbohydrate active enzymes^37^. Such a function would not necessarily imply that the aromatic residues interact with the polymer chain that is being deacetylated.

Analysis of charge distributions on the protein surface show that *An*CDA has a surplus of negative charge in a region that could interact with the non-reducing end of a longer oligomeric substrate (Fig. 8). It is conceivable that extensive electrostatic interactions between this negative surface and a positively charged, almost completely deacetylated, substrate explain why the enzyme slowly deacetylates the reducing ends of chito-oligomers. This distribution differs from the combined positive-negative distribution that is observed in the *Sp*PgdA catalytic centre (Fig. 8). *Sp*PgdA is a peptidoglycan de-*N*-acetylase that has evolved to accommodate negative charges on the *N*-acetyl muramic acids residues in peptidoglycan; such negative charges lack in chitin. The surface charge of *An*CDA is special compared to other deacetylases as well; *Cl*CDA shows a more negative surface charge on the positive side of the catalytic cleft compared to *An*CDA. It is interesting to note the differences between the CDAs, which may suggest different functionalities.

### Concluding remarks

*An*CDA was expressed as soluble protein in *E. coli* and is stable and easy to produce, meaning that it is a potentially useful enzyme for industrial applications. As discussed above, *An*CDA seems to have broader substrate specificity than other characterized members of the CE4 family and the enzyme seems well suited to act on chitin and its partially deacetylated forms. Indeed, the enzyme was highly active on chitosan and on chitin that had been “de-crystallized” by CBP21. The crystal structure of *An*CDA shows that the enzyme has an open active-site structure with seemingly few subsites, and a hydrophobic surface patch that might be involved in substrate binding. The aligned structures of *An*CDA and *Vc*CDA with a chitotriose bound show that the two enzymes have similar active sites but shows that *An*CDA has some interesting features in subsite +1, similar to one of the best characterized CDAs so far, *Cl*CDA^9, 22^. Another interesting feature of *An*CDA is the negative surface charge in the part of the enzyme that interacts with the non-reducing end of the substrate, and which is possibly involved in interactions with positively charged (i.e. deacetylated) sugar units. In-depth analysis of product formation showed that the reducing end of chito-oligomers is only very slowly deacetylated, whereas the non-reducing end is more prone to deacetylation. This underpins the importance of the +1 subsite, which indeed seems to interact more strongly with the substrate compared to the −1 subsite.

Despite these novel insights, questions concerning the biological function of these CE4 enzymes remain unanswered: What is their natural substrate? What is their biological function? What are the structural determinants of the variation in substrate specificity observed when comparing data for various CDAs? In a recent report, Andrés *et al*.^18^ proposed a “substrate capping model”, implying that substrate specificity is defined by the absence and presence of different loops near the catalytic center^18^. Andrés *et al*. studied *Vc*CDA, which contains several large extensions relative to *An*CDA, and which strictly deacetylates the sugar next to the non-reducing end^38^, which is different from *An*CDA that deacetylate several positions on a chito-oligomer. Compared to *Vc*CDA, and a similar protein from *Vibrio parahaemolyticus* ^24^, *An*CDA, and other CDAs^9, 15, 17^, have smaller loops resulting in a more open active site. Hence, *An*CDA is able to bind a chito-oligomer in different manners and can catalyse multiple deacetylations at various positions in the substrate.

## Materials and Methods

### Strains and culture conditions

*Aspergillus nidulans* FGSC A4 (obtained from the Fungal Genetics Stock Center, Kansas City, Kansas City, MO, USA) was grown at 37 °C in solid YAG medium (5 g/L yeast extract, 20 g/L dextrose, 20 g/L agar, 1 ml/L Cove’s Trace Elements (0.04 g/L Na_2_B_4_O_7_:10H_2_O, 0.4 g/L CuSO_4_:_5_H_2_O, 0.8 g/L FeCl_3_, 0.8 g/L MnSO_4_:H_2_O, 0.8 g/L NaMoO_4_:2H_2_O, 8 g/L ZnSO_4_:7H_2_O), 1.2 g/L MgSO_4_·7H_2_O) for 24–48 h to provide an inoculum, and then in liquid YG medium (=YAG medium without agar) for16–24 h with vigorous shaking/aeration (250–300 rpm)^39^. Genomic DNA was isolated with the SP Fungal DNA Mini Kit (Omega Bio-tek, Norcross, GA, USA). As the host cell for DNA transformation and protein expression, *Escherichia coli* TOP10 (Invitrogen, Carlsbad, CA, USA) was grown in 2 × TY medium (16 g/L Tryptone, 10 g/L yeast extract, 5 g/L NaCl) containing 100 mg of ampicillin per liter.

### Cloning, expression and purification of AnCDA

The gene (EAA66447) was amplified from *Aspergillus nidulans* FGSC A4 genomic DNA using overlap extension polymerase chain reactions to remove the 2 introns and the following primers, synthesized at Eurofins MWG Operon (Ebersberg Germany): P1f (*Bgl*II): cga aga tct acg cct ctg cct ttg gtt c; P2r: gag acg tgg tcg tat gta tgt gcg ccg act tga tg; P3f: caa gtc ggc gca cat aca tac gac cac gtc tcc ctc c; P4r: cca aca gtc gta gct atc aac cct cga gca tta ac; and P5r (*Hind*III): cag aag ctt tca atg ata cca cgc aat ctc tcc atc acc gag aca atc acc aac agt cgt agc tat caa c. *Bgl*II and *Hind*III sites were incorporated at the start and the end of the *An*CDA gene, respectively, to produce an in-frame N-terminal His tag-fused construct in the pBAD/HisB(s) vector. This vector is a variant of the commercial vector pBAD/HisB (Invitrogen, CA, USA) containing a shortened region between the N-terminal polyhistidine tail and the down-stream multiple cloning site^40^. In the final construct, expression of the gene was under the transcriptional control of the arabinose-regulated *araBAD* promoter. The gene product consists of the sequence MAHHHHHHHRS followed by the mature *An*CDA (i.e. *An*CDA without its 18-residue N-terminal signal peptide). The resulting expression vector was transformed into *E. coli* TOP 10 and the correctness of the gene fragment was verified by BigDye® Terminator v3.1 cycle sequencing using an in-house sequencing facility.

For protein expression, a transformant was cultured at 37 °C in 2 × TY medium containing 100 mg of ampicillin per liter until the OD_600_ reached 0.6, after which gene expression was induced by adding 0.02% (w/v; final concentration) arabinose. After overnight incubation at 28 °C, cells were harvested by centrifugation and the protein was purified to homogeneity by affinity column chromatography using a Ni-NTA Superflow Column (Qiagen, Venlo, The Netherlands). Protein concentrations were determined using the Bio-Rad Protein Assay (Bio-Rad, CA, USA), with bovine serum albumin as a standard. After buffer exchange, using an Amicon® Ultra Centrifugal Filter (MW10,000, Millipore), to 20 mM Tris pH 8.0, with 20 mM NaCl, the protein was stored at −20 °C in 20 mM Tris pH 8.0, 20 mM NaCl, and 50% glycerol. All experiments in this study, including crystallization (see below), were done with protein containing the short N-terminal His-tag, which was not considered a problem since the N-terminus is located on the opposite side of the protein, relative to the catalytic center.

### Preparation of substrates for deacetylation assays

*N*-acetylchito-oligomers [(GlcNAc)_1–6_] were purchased from Megazyme (Wicklow, Ireland). Shrimp (*Pandalus borealis*) shell α-chitin from Hov-Bio (Tromsø, Norway) and squid pen β-chitin from France Chitin (Marseille, France) were sieved with 75 μm mesh. As an alternative source of β-chitin we used squid pen chitin purchased from Yaegaki (Hayashida, Japan) exposed to cutter milling for 60 sec for particle size reduction (See ref. 41 for methods). Soluble chitosans with a degree of *N*-acetylation of 61% and 64% (F_A_ 0.61 and 0.64), with a random distribution of *N*-acetylated and de-*N*-acetylated units^31^, were a gift from Professor Kjell M. Vårum from the Department of Biotechnology, Norwegian University of Science and Technology. Acetylated glucuronoxylan with an approximate mass distribution from 500 Da to 2500 Da (roughly estimated from MALDI-ToF MS analyses, see below) was prepared as described previously^42^. Acetylated xylan was prepared by steam explosion (190 °C, 10 min) of bagasse adjusted with succinate to pH 4.0, which yielded a soluble fraction containing acetylated xylan with an approximate mass distribution from 500 Da to 3500 Da. The necessary ultrafiltration was conducted using a two-step procedure with a Millipore UF system, first running a 10 kDa cutoff filter (Millipore) and then running the permeate from the first step on a 1 kDa cutoff filter (Millipore). Peptidoglycan from *Streptomyces sp*. and all other chemicals were purchased from Sigma (St Louis, MO).

### Enzyme Assays

Standard reaction mixtures of 100 µL containing 40 nM *An*CDA, 10 µM CoCl_2_, 50 mM Tris pH 8.0 and substrate were incubated at 37 °C for 15 min and reactions were quenched by addition of acetonitrile to a final volume of 50% (v/v). Experiments were performed in triplicate and corrected for background from control reactions without enzyme. Acetate release was measured by IC as described in the IC section below. Substrate concentrations varied between substrates and are listed in Table 1. For determination of *K*_m_ and *k*_cat_, (GlcNAc)_5_ was used as substrate at concentrations ranging from 0.05 mM to 2 mM. Reactions were performed as above with 15 min incubation time. The data were processed using GraphPad Prism 6.03®.

For the experiment with CBP21, a chitin-active lytic polysaccharide monooxygenase from *Serratia marcescens*, 1 mg/mL cutter milled β-chitin was incubated in 50 mM Tris pH 8.0 with 1 µM *An*CDA, 1 µM CBP21 or 1 µM of each enzyme, 250 µM CoCl_2_ and 1 mM ascorbic acid, at 37 °C. Reactions were terminated by addition of acetonitrile to a final volume of 50% (v/v) and acetate was analysed as described in the IC section below. All measurements were corrected for background acetate release by subtraction of signals obtained for control reactions without enzyme.

### Effects of pH, temperature and metal ions

Reactions were performed at 30–100 °C under otherwise standard conditions to determine the temperature optimum for activity, and performed at pH 3.0–10.0 under otherwise standard conditions to determine the pH optimum for activity. Buffers used were 50 mM citrate-phosphate (pH 3.0–7.0), 50 mM Tris-HCl (pH 8.0), and 50 mM boric acid (pH 9.0–10.0). To determine stability, the enzyme was pre-incubated in buffers of pH 3.0–10.0 or at different temperatures (30–100 °C; pH 8.0) for one hour. Subsequently, remaining enzyme activity was measured using the standard activity assay. The pre-incubation at varying pH was done at high enzyme concentration and the subsequent pH adjustment was achieved by diluting in standard reaction buffer. The effect of metal ions was tested by adding 10 μM of ions to the reaction mixtures.

### Ion chromatography (IC)

Acetate release was measured by IC using a Dionex ICS3000 system with suppressed conductivity detection. The separation was obtained at 0.375 mL/min on a Dionex IonPac organic acid column AS11-HC (2 × 250 mm analytical) with an AG11-HC (2 × 50 mm guard) with the following with the following elution scheme: 0–8 min, 1 mM KOH; 8–17 min, 1–30 mM KOH; 17–19 min, 30–60 mM KOH; 19–24 min, 60 mM KOH; 24–25 min, 60–1 mM KOH; 25–31 min, 1 mM KOH. Operation of the IC and processing of chromatograms were performed using the Chromeleon 7 software (Dionex Corp.).

### MALDI-ToF-MS

For sequence analysis of chito-oligomers, reductive amination of hetero-chito-oligomers with 2-aminoacridone (AMAC) was performed as previously described^28^. For subsequent sequence analysis, 1 μL containing 1–3 nmol of oligosaccharides was mixed with 2 μL of matrix solution (15 mg/mL 2,5-Dihydroxybenzoic acid), spotted on a 384-Spot MALDI Plate, and dried at room temperature. MS spectra were acquired using an Ultraflex™ ToF/ToF mass spectrometer (Bruker Daltonik GmbH, Bremen, Germany) as described previously^43^.

### Structure determination

The pBAD-*An*CDA plasmid was transformed into BL21 (DE3:pLysS) cells for expression of protein used in crystallographic experiments. Cells were grown to OD_600_ = 0.6 in LB medium containing 50 μg/mL ampicillin, at which time protein expression was induced with L-arabinose (final concentration = 0.02%, w/v). After incubation for 36 hours at 18 °C, the cells were pelleted and suspended in buffer containing 25 mM Tris at pH 7.5 and 150 mM NaCl. The cells were lysed by continuous cell disruption (Constant Systems Ltd. Northants, UK) and the lysate was clarified by centrifugation at 40,000 *g* for 1 hour at 4 °C. The His-tagged *An*CDA protein was isolated using a HiTrap IMAC FF column (GE Healthcare, Little Chalfont, UK) and further purified by ion-exchange (HiTrap Q FF; GE Healthcare) and size exclusion chromatography (Superdex 75 26/60; GE Healthcare). The purified protein was concentrated by centrifugation in 25 mM Tris at pH 7.5 to 10 mg/mL using a Vivaspin 10 kDa spin concentrator, and plated in MRC sitting-drop plates (Molecular Dimensions, Newmarket, UK) using mother liquor containing 0.1 M NaH_2_PO_4_, 5% hexanediol, and 20% PEG 3350. Crystals appeared within 18–24 hours.

Data was collected at beamline I02 at the Diamond Light Source (Didcot, UK). Image files were indexed, processed and integrated using Mosflm^44^ and scaled using Scala implemented in the CCP4 suite of programs^45^. Initial phases were calculated by molecular replacement using the structure for a chitin deacetylase from *Colletotrichum lindemuthianum* (*Cl*CDA), PDB accession code 2IW0^9^, and the program Molrep^46, 47^. Manual model building and refinement were performed using Coot^48^ and Refmac5^49^, respectively. Refinement statistics can be found in Table 2. The final model was validated with Procheck^50^.
